# Determinants of Participants’ Follow-Up and Characterization of Representativeness in Flu Near You, A Participatory Disease Surveillance System

**DOI:** 10.2196/publichealth.7304

**Published:** 2017-04-07

**Authors:** Kristin Baltrusaitis, Mauricio Santillana, Adam W Crawley, Rumi Chunara, Mark Smolinski, John S Brownstein

**Affiliations:** ^1^ Computational Health Informatics Program Boston Children’s Hospital Boston, MA United States; ^2^ Department of Biostatistics Boston University School of Public Health Boston, MA United States; ^3^ Harvard Medical School Boston, MA United States; ^4^ Harvard School of Engineering and Applied Sciences Cambridge, MA United States; ^5^ Skoll Global Threats Fund San Francisco, CA United States; ^6^ The Global Institute of Public Health New York University New York, NY United States; ^7^ Computer Science & Engineering New York University New York, NY United States

**Keywords:** public health surveillance, influenza, human, community-based participatory research, crowdsourcing, public health informatics, digital disease detection

## Abstract

**Background:**

Flu Near You (FNY) is an Internet-based participatory surveillance system in the United States and Canada that allows volunteers to report influenza-like symptoms using a brief weekly symptom report.

**Objective:**

Our objective was to evaluate the representativeness of the FNY population compared with the general population of the United States, explore the demographic and behavioral characteristics associated with FNY’s high-participation users, and summarize results from a user survey of a cohort of FNY participants.

**Methods:**

We compared (1) the representativeness of sex and age groups of FNY participants during the 2014-2015 flu season versus the general US population and (2) the distribution of Human Development Index (HDI) scores of FNY participants versus that of the general US population. We analyzed associations between demographic and behavioral factors and the level of participant follow-up (ie, high vs low). Finally, descriptive statistics of responses from FNY’s 2015 and 2016 end-of-season user surveys were calculated.

**Results:**

During the 2014-2015 influenza season, 47,234 unique participants had at least one FNY symptom report that was either self-reported (users) or submitted on their behalf (household members). The proportion of female FNY participants was significantly higher than that of the general US population (n=28,906, 61.2% vs 51.1%, *P*<.001). Although each age group was represented in the FNY population, the age distribution was significantly different from that of the US population (*P*<.001). Compared with the US population, FNY had a greater proportion of individuals with HDI >5.0, signaling that the FNY user distribution was more affluent and educated than the US population baseline. We found that high-participation use (ie, higher participation in follow-up symptom reports) was associated with sex (females were 25% less likely than men to be high-participation users), higher HDI, not reporting an influenza-like illness at the first symptom report, older age, and reporting for household members (all differences between high- and low-participation users *P*<.001). Approximately 10% of FNY users completed an additional survey at the end of the flu season that assessed detailed user characteristics (3217/33,324 in 2015; 4850/44,313 in 2016). Of these users, most identified as being either retired or employed in the health, education, and social services sectors and indicated that they achieved a bachelor’s degree or higher.

**Conclusions:**

The representativeness of the FNY population and characteristics of its high-participation users are consistent with what has been observed in other Internet-based influenza surveillance systems. With targeted recruitment of underrepresented populations, FNY may improve as a complementary system to timely tracking of flu activity, especially in populations that do not seek medical attention and in areas with poor official surveillance data.

## Introduction

Influenza infections are associated with thousands of deaths each year in the United States [1]. Reliable real-time estimates of the temporal and geographical trends of the spread of influenza in the population are crucial to prepare for unusual influenza epidemics, for clinical resource allocation, and to assess vaccine effectiveness. In the United States, the Centers for Disease Control and Prevention (CDC) collects, compiles, and analyzes data from laboratories, outpatient health care offices, mortality surveillance systems, hospitals, and state health departments, and summarizes these datasets in weekly influenza surveillance reports. However, there is typically a 2-week lag between time of illnesses and publication of these reports. Furthermore, because these data sources report only on those individuals who seek medical care, an unknown proportion of individuals who do not visit health care facilities are unrepresented.

Over the past decade, Internet-based biosurveillance systems have developed as a way to provide an informal, complementary approach to traditional (syndromic) surveillance methods. These systems have the potential to reach a wider population and provide real-time access to users’ symptom reports because they leverage alternative data sources, such as Google [2], Yahoo [3], and Baidu [4] Internet searches; Twitter posts [5-7]; Wikipedia article views [8,9]; and clinicians’ database queries [10]. Internet search-based efforts in particular (ie, methods that use patterns of flu-related Internet searches to track flu) have led to very accurate Internet flu tracking systems [11-13]. Crowdsourced Internet-based participatory syndromic surveillance programs, such as Influenzanet in Europe, FluTracking in Australia, and Flu Near You (FNY) in the United States and Canada, have also been developed to track community influenza activity. These systems correlate well with traditional, clinical-based influenza-like illness (ILI) activity surveillance tools [14-17], and other platforms, such as GoViral, have validated the use of participatory information for disease surveillance by comparing volunteers’ self-reported symptoms with specimens [18]. Although participatory surveillance systems track flu activity in a timely fashion, a large, diverse cohort of users who participate regularly and are representative of the population is essential for these systems to work effectively. Current surveillance systems have made efforts to quantify the biases between participant and general populations and investigate factors that influence follow-up participation [19-22].

We focused on FNY, which is administered by HealthMap of Boston Children’s Hospital in partnership with the Skoll Global Threats Fund [23]. Specifically, we evaluated the representativeness of the FNY participant population compared with the general population of the United States, explored the demographic and behavioral characteristics that are associated with FNY’s high-participation users, and summarized the results from a survey of a cohort of FNY users.

## Methods

### Representativeness of Participant Population

#### FNY Participants

Any resident of the United States or Canada can register as a user through the FNY website [24], mobile app, or Facebook. Upon registration, users provide information on their sex, date of birth, residential zip code, and email address. Although individuals must be at least 13 years of age to register, users can also add household members of any age and submit reports on their behalf. In exchange for participating in FNY, users can visualize local flu activity on maps, connect with local public health organizations, and find nearby locations offering flu vaccines. In this paper we define users as the population of individuals who registered with FNY and participants as the combined population of users and household members.

#### FNY Data Collection

Following registration, FNY users are asked to submit brief weekly reports where they can report any symptoms that they or any registered household members had during the previous week (Monday through Sunday). The symptoms in the report include fever, cough, sore throat, shortness of breath, chills or night sweats, fatigue, nausea or vomiting, diarrhea, headache, and body ache. If a user did not have any of these symptoms, he or she can also choose “I did not have any of the listed symptoms.” However, if a user reports any of these symptoms, he or she is asked to provide the date of symptom onset. In addition, users are asked if they have received an influenza vaccination for the current flu season. Users are sent a reminder to complete the symptom report every Monday through either an email with a survey link or a push notification on their mobile phone. Although data are collected throughout the year, users have the option to suspend symptom reporting during the summer.

#### Census and Social Data Collection

We obtained national estimates of sex and age from the United States Census Bureau’s 2014 annual estimates of resident population [25]. Socioeconomic status was estimated at the county level using the Human Development Index (HDI) as a proxy [26]. The HDI is the county-level average of the educational index and income index and is measured on scale from 0-10, where 0 is the lowest HDI score and 10 is the highest. The educational index is a weighted average of the educational attainment index (ie, the measure of the overall level of educational attainment achieved by the adult population) and the enrollment index (ie, total number of students enrolled in school, of any age at any level, divided by the total school-aged population of 3- to 24-year-olds, inclusive). We calculated the income index from county-level median income [26]. The use of county-level HDI as a proxy for socioeconomic status was further assessed by estimating user-specific HDI from the cohort survey results (see below) and comparing these estimates with the corresponding county-level HDI estimate. Consistent with the method established by the Measure of America, the income index of the user-specific HDI was estimated by dividing the difference between the log of the zip code-level median income of the user and the log of the minimum US median income by the difference between the log of the maximum and the log of the minimum US median incomes, and then multiplying this ratio by 10 to scale the index between 0 and 10 [27]. We estimated the educational index from the level of education response of the user survey, where we assigned smaller values to lower educational attainment, such as “did not graduate high school,” than to higher educational attainment, such as a doctoral, law, or medical degree.

#### Comparison of FNY Participant Population With the US Population

Although FNY includes participants from the United States, Canada, and US territories, such as Puerto Rico, the majority of users reside within the 50 US states. As a result, the participant population used in this analysis included all registered users and household members residing within the 50 US states with complete sex, date of birth, and zip code information who submitted at least one symptom report during the 2014-2015 flu season, as defined by CDC weeks 40 (week ending October 4, 2014) through 20 (week ending May 23, 2015). We addressed the representativeness of sex (male and female) and age groups (<5, 5-14, 15-29, 30-39, 40-49, 50-59, 60-69, 70-79, ≥80 years) of FNY participants compared with the general US population using a 2-sided chi-square goodness-of-fit test. We compared the county-level distribution of HDI scores of FNY participants with that of the general US population using a 2-sample Kolmogorov-Smirnov test. For sensitivity purposes, we also performed these calculations using FNY data from the 2012-2013 and 2013-2014 flu seasons.

### Characteristics of High-Participation Users

For this analysis, we considered only users who reported their own information, completed at least one symptom report during the 2014-2015 flu season during or before CDC week 17, and provided sex information at registration. In addition, we chose only residents of the United States between ages 13 and 80 years at their registration date because users must be at least 13 years of age to register. A limit of 80 years of age was used to account for possible errors in date of birth input at user registration. Users who met these criteria were classified as either a high-participation user or a low-participation user based on the number of symptom reports they submitted during the 2014-2015 flu season. Users who completed more than 3 symptom reports during the 2014-2015 flu season were identified as high-participation users.

The demographic factors used in this analysis were sex (male or female), age group (13-29, 30-39, 40-49, 50-59, 60-69, and 70-79 years), and HDI as a continuous variable (see above for a description of methods used to calculate HDI). In addition, we included whether or not an ILI, as defined by the CDC, was reported at first entry. Although we did not examine information from individual household members in the analysis, we also included whether or not primary participants reported on behalf of other household members.

We analyzed associations between these demographic and behavioral factors and the level of participant follow-up using multivariable logistic regression. For odds ratio (OR) comparisons among age groups, we used 50-59 years as the reference group because it had the largest number of users. The demographic and behavioral factors were treated as independent variables, while level of follow-up was a dichotomous outcome (high-participation user vs low-participation user). We dichotomized the outcome because the distribution of number of reports was not normally distributed, and we determined the cutoff value of 3 empirically by assessing the histogram of number of reports. Sensitivity analyses confirmed the robustness of our findings. These additional analyses were conducted using more- and less-stringent definitions of high-participation users—specifically, more than 10 entries and more than 1 entry, respectively—for the 2012-2013, 2013-2014, and 2014-2015 flu seasons. Data were analyzed using R for Mac OS X version 3.1.1 (R Foundation).

### Cohort Survey

To supplement data from user registrations, the FNY team conducted end-of-season user surveys in June 2015 and May 2016. The surveys were administered through SurveyGizmo online survey software (Widgix LLC), with survey invitations sent via email to all active FNY users. A completed survey entered the user into a raffle for incentives that included an iPad and US $100 gift cards. Users were asked a variety of questions designed to better understand their interest in and motivations for reporting to FNY. Here we report on responses to a subset of questions (Textbox 1).

**Subset of survey questions administered to users of Flu Near You.**

- How did you first hear about Flu Near You?
- Why did you sign up for Flu Near You?
- What is your primary motivation for continuing to use Flu Near You?
- Do you consider yourself more or less likely to report to Flu Near You when you have symptoms of illness to report?
- When you report to Flu Near You, do you report on any symptoms you’ve experienced during the week, or only symptoms that you think may be linked to the flu?
- What symptoms do you think are the primary symptoms of a flu infection?
- What is your industry/occupation?
- What is your level of education?

We exported survey responses to Excel 2016 (Microsoft Corporation) and Stata version 13 (StataCorp LP) for analysis and tabulation of descriptive statistics.

## Results

### Representativeness of Participant Population

**Figure 1:**
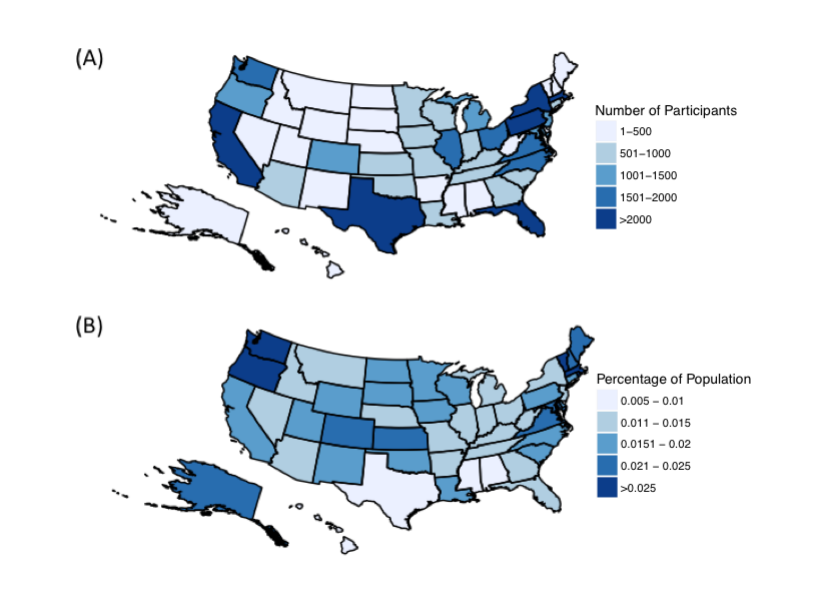
Distribution of participants for 2014- 2015 flu season by state (A) unadjusted distribution (B) adjusted distribution by state population.

Among states, California had the largest number of participants for the 2014-2015 flu season (n=6595), while Wyoming had the fewest (n=89) (see Figure 1). When we adjusted for state population size, Rhode Island had the greatest per capita representation (0.04%), and Mississippi had the lowest (0.008%) (Figure 1). The 2012-2013 and 2013-2014 flu seasons displayed a similar geographic distribution.

**Figure 2:**
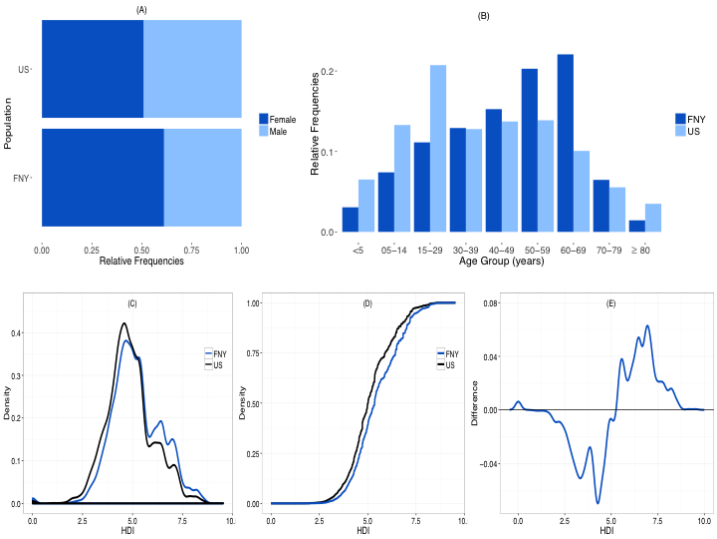
(A) Sex and (B) age profiles of Flu Near You (FNY) participants and comparison with the general population. (C) Distribution and (D) cumulative distribution of Human Development Index (HDI) scores by county for FNY and US populations and (E) difference between US and FNY distributions.

During the 2014-2015 influenza season, 47,234 unique participants had at least one symptom report that was either self-reported or submitted on their behalf. Of these participants, 28,906 (61.20%) were female and 18,328 (38.80%) were male. The proportion of female FNY participants was significantly overrepresented when compared with the general US population (51.1% female, *P*<.001) (Figure 2 A). Although each age group was represented in the FNY population, the distribution of age was significantly different from that of the US population (*P*<.001; data not shown). Overall, adult populations were overrepresented (ages 40-79 years), and both younger populations (ages <30 years) and older populations (ages ≥80 years) were underrepresented (Figure 2 B). The HDI range in the FNY population was 0-9.54 with a median of 5.03. As Figure 2 C and Figure 2 D show, the distribution of HDI scores was significantly different between the FNY population and the US population (*P*<.001). In general, FNY had a greater proportion of individuals with HDI scores greater than 5.0 (Figure 2 E). These descriptive statistics were similar across all 3 flu seasons (2012-2013, 2013-2014, and 2014-2015).

### Characteristics of High-Participation Users

**Figure 3:**
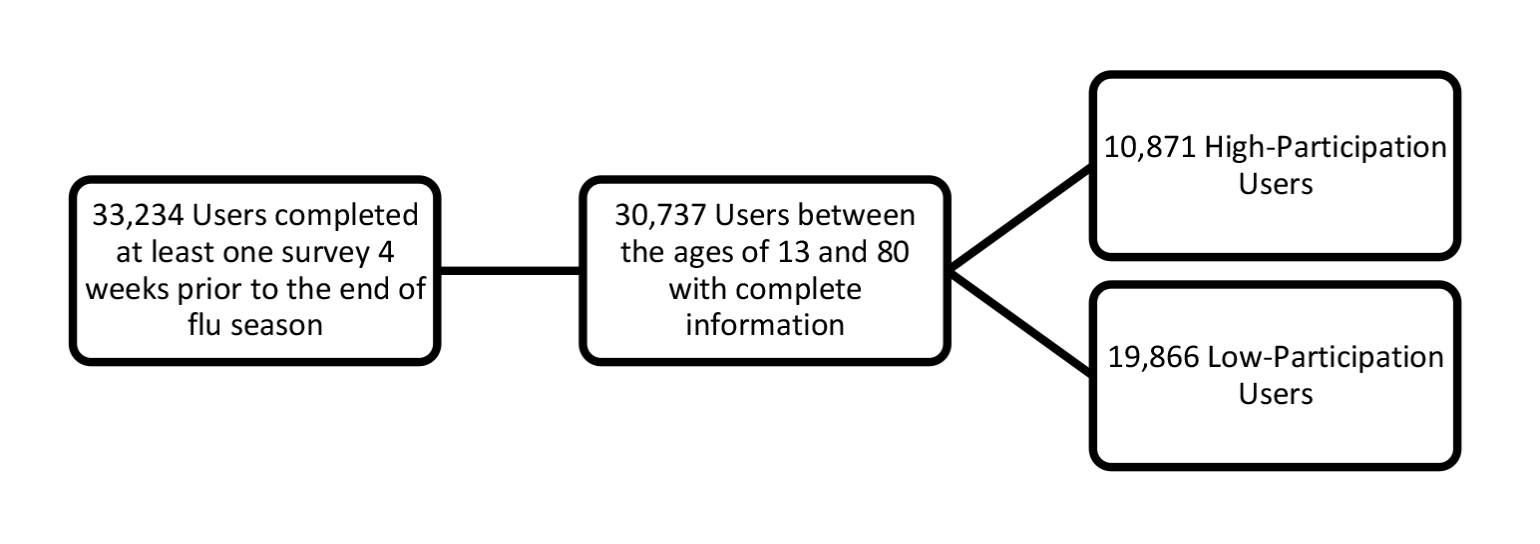
Flowchart of user enrollment.

Figure 3 shows a flowchart of FNY user enrollment, including the number of users classified as either a high-participation user or a low-participation user. Table 1 summarizes results (adjusted ORs). Overall, females were 25% less likely than males to be high-participation users (*P*<.001). Users who reported for additional household members had 3.29 times the odds of being high-participation users compared with users who did not report for additional household members (*P*<.001). Each unit increase in HDI was also associated with an increase in the odds of being a high-participation user (OR 1.12, *P*<.001). Users who reported symptoms meeting the definition of ILI at the first entry were 78% less likely than those who did not to be high-participation users (*P*<.001). There was a significant difference in participation among age groups (*P*<.001). In general, the odds of being a high-participation user increased with age.

**Table 1:** Summary of adjusted odds ratios (ORs) of being a high-participation user of Flu Near You.

Variable		Reference group	Adjusted OR	95% CI	*P* value
Sex: female		Male	0.75	0.71-0.79	<.001
Household members: yes		No	3.29	3.12-3.35	<.001
Human Development Index		N/A^a^	1.12	1.09-1.14	<.001
Influenza-like illness status at first survey: yes		No	0.22	0.19-0.25	<.001
**Age group (years)**					
	13-29	50-59	0.67	0.61-0.74	<.001
	30-39	50-59	0.54	0.49-0.58	<.001
	40-49	50-59	0.70	0.64-0.75	<.001
	60-69	50-59	1.14	1.07-1.23	<.001
	70-79	50-59	1.23	1.11-1.36	<.001

^a^N/A: not applicable.

### Cohort Survey

In 2015 the FNY user survey received 3217 responses from users, and the total numbers of users responding to the survey increased to 4850 in 2016. In both years the largest proportion of users identified as being retired (878/3217, 27.29%, in 2015; 1651/4850, 34.04%, in 2016), followed by users employed in the fields of health care and social assistance (620/3217, 19.27%, in 2015; 902/4850, 18.59%, in 2016), professional, scientific, and technical services (370/3217, 11.50%, in 2015; 453/4850, 9.34%, in 2016), and educational services (309/3217, 9.61%, in 2015; 409/4850, 8.43%, in 2016). These 4 categories accounted for 67.67% (2177/3217) of survey respondents in 2015 and 70.41% (3415/4850) in 2016. These results suggest that FNY relies heavily on retirees and those employed in the health, education, and social services sectors.

**Figure 4:**
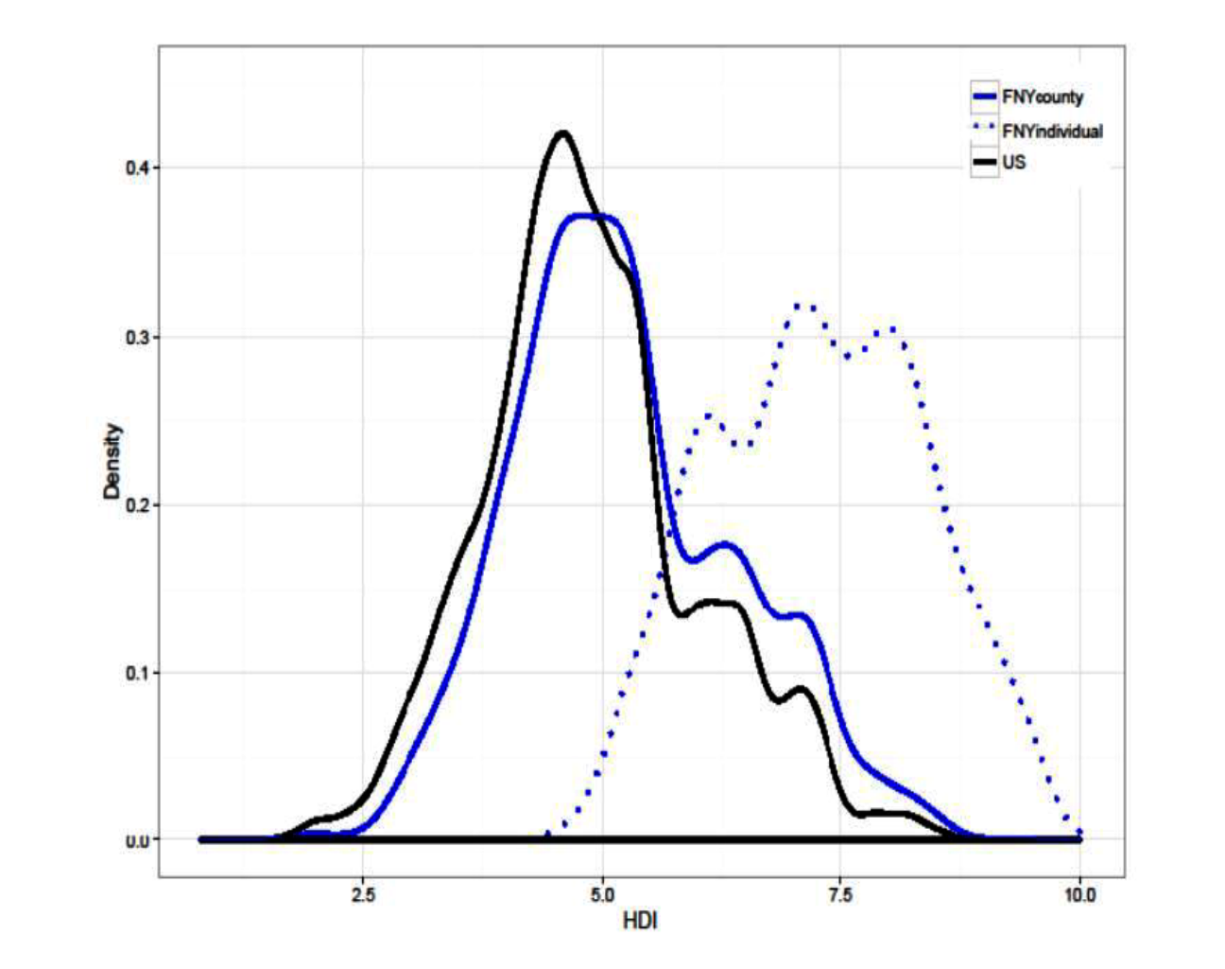
Distributions of county-level Human Development Index (HDI) scores and individual user-specific HDI scores in Flu Near You (FNY) survey participants.

In addition, our user surveys indicated that the majority of respondents had achieved a bachelor’s degree or higher (2322/3217, 72.18%, in 2015; 3315/4850, 68.35%, in 2016), while less than 1% had not graduated high school. Approximately one-quarter of respondents had attained a master’s degree in each survey year (845/3217, 26.27%, in 2015; 1181/4850, 24.35%, in 2016), while some respondents held doctoral or other advanced degrees (435/3217, 13.52%, in 2015; 602/4850, 12.41%, in 2016). These results support trends seen in participants’ HDI scores, suggesting that the FNY participant population may have a higher educational attainment than the general US population (Figure 4).

## Discussion

Our results, in combination with previous work [15], show that FNY has the potential to achieve its goals, which include collecting and sharing participant-reported symptoms in order to increase awareness of ILI activity, generate early signals of ILI occurrence, and track ILI symptoms across the United States. Here, we show that participation varied across geographic location, sex, age, and HDI. Although all 50 states were represented during the 2014-2015 flu season, a few states had fewer than 500 participants, and the geographic distribution shows large gaps of information, especially in areas in the Midwest and South. FNY participants tended to cluster around large urban areas, with especially large user bases in the greater metropolitan areas surrounding Boston, New York City, and San Francisco.

Overall, females were overrepresented in our participant population (61.20%). This overrepresentation is consistent with findings from other participatory surveillance systems. During the 2011-2012 flu season, Influenzanet participants were more likely to be female than in the general population (56.8% vs 50.9%, *P*<.001), and among FluTracking participants who completed at least one survey each year, 66% in 2011 and 64% in 2012 were female [19,28]. This overrepresentation of female participants is reflective of other studies showing that women are more likely than men to seek online health information [29,30].

FNY participants also had a higher HDI than that of the US population. This finding aligns with the results of our user survey conducted in 2015 and 2016. When comparing county-level HDI estimates with the user-specific HDI estimates within the population of FNY users who completed the 2016 survey, we found that for most survey participants, the county-level HDI underestimated the user-specific HDI, which further supports our initial findings that FNY participants have a higher HDI than the US population. These relatively high levels of HDI in the FNY population can be in part due to patterns in Internet penetration. Studies from the Pew Research Center have shown that Americans with high education levels and those in relatively affluent households have high Internet penetration [31].

Nor was the FNY population representative of the general US population in terms of age. Both younger populations (ages <30 years) and older populations (ages ≥80 years) were underrepresented, while the age groups between 40 and 80 years were overrepresented. As with sex, patterns of age representations were similar for both Influenzanet and FluTracking participants [19,28]. All of these differences in population characteristics (ie, sex, HDI, and age) were consistent across all 3 flu seasons we assessed.

**Figure 5:**
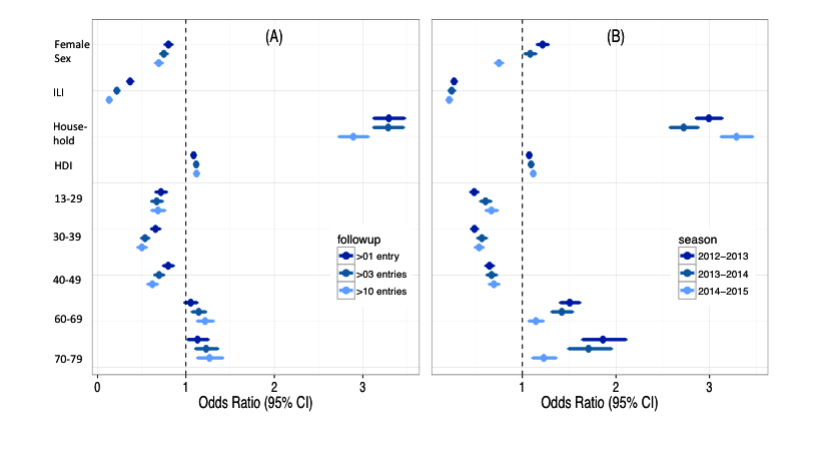
Odds ratios of high-participation users, by sex, influenza-like illness (ILI), Human Development Index (HDI), and age range, for (A) different levels of follow-up during the 2014-2015 flu season and (B) the 2012-2013, 2013-2014, and 2014-2015 flu seasons using more than 3 entries to define high-participation users.

We found that higher participation in follow-up symptom reports was associated with sex (male), reporting for household members, higher HDI score, not reporting an ILI at the first survey, and older age. These findings were consistent using both more-stringent (>10 entries) and less-stringent (>1 entry) definitions of good users (Figure 5 A). The results were consistent across all 3 seasons (Figure 5 B), except for sex. While females were less likely to be better users during the 2014-2015 season, this was not the case during the 2012-2013 and 2013-2014 seasons. Given the differences in reporting patterns by sex across years, an underlying factor, such as method of member recruitment, may be a confounder of this association. In addition, the ORs comparing participation habits between males and females were close to 1 (Figure 5 B), and a previously published study from Influenzanet found that there were no significant differences between reporting for males and females [21].

The biases intrinsic within the FNY population are consistent with biases found in other Internet-based influenza surveillance systems. Despite these biases in the sociodemographic characteristics of the population, previous studies have shown that FNY and traditional disease surveillance systems capture similar trends of ILI rates at the national level [12,15]. The CDC has established a flu surveillance system that is robust and well accepted, by tracking individuals with ILI symptoms who seek medical care. Because only 35% of FNY users who report ILI symptoms seek medical attention, FNY captures flu activity in populations not accounted for by official surveillance data. As a result, with targeted recruitment, FNY may become a robust and complementary surveillance system that will benefit public health officials and the general population.

## Abbreviations

CDC: Centers for Disease Control and Prevention
FNY: Flu Near You
HDI: Human Development Index
ILI: influenza-like illness
OR: odds ratio
